# Comparing the new Ifakara Ambient Chamber Test with WHO cone and tunnel tests for bioefficacy and non-inferiority testing of insecticide-treated nets

**DOI:** 10.1186/s12936-019-2741-y

**Published:** 2019-04-30

**Authors:** Dennis J. Massue, Lena M. Lorenz, Jason D. Moore, Watson S. Ntabaliba, Samuel Ackerman, Zawadi M. Mboma, William N. Kisinza, Emmanuel Mbuba, Selemani Mmbaga, John Bradley, Hans J. Overgaard, Sarah J. Moore

**Affiliations:** 1 Epidemiology and Public Health Department, Swiss Institute of Tropical and Public Health, Soccinstrase 57, 4002 Basel, Switzerland; 2 University of Basel, Petersplatz 1, 4003 Basel, Switzerland; 3 National Institute for Medical Research, Amani Research Centre, P. O. Box 81, Muheza, Tanga, Tanzania; 4 Ifakara Health Institute, P. O. Box 74, Bagamoyo, Pwani, Tanzania; 5 Department of Disease Control, Faculty of Infectious and Tropical Diseases, London School of Hygiene and Tropical Medicine, London, WC1E 7HT UK; 6 MRC Tropical Epidemiology Group, London School of Hygiene and Tropical Medicine, London, WC1E 7HT UK; 7 Faculty of Science and Technology, Norwegian University of Life Sciences, Ås, Norway

**Keywords:** Biological efficacy, WHO cone test, WHO tunnel test, I-ACT, Ifakara Ambient Chamber Test, Long lasting insecticidal nets, Durability, Non-inferiority

## Abstract

**Background:**

Insecticide-treated net (ITN) durability, measured through physical integrity and bioefficacy, must be accurately assessed in order to plan the timely replacement of worn out nets and guide procurement of longer-lasting, cost-effective nets. World Health Organization (WHO) guidance advises that new intervention class ITNs be assessed 3 years after distribution, in experimental huts. In order to obtain information on whole-net efficacy cost-effectively and with adequate replication, a new bioassay, the Ifakara Ambient Chamber Test (I-ACT), a semi-field whole net assay baited with human host, was compared to established WHO durability testing methods.

**Methods:**

Two experiments were conducted using pyrethroid-susceptible female adult *Anopheles gambiae* sensu stricto comparing bioefficacy of Olyset^®^, PermaNet^®^ 2.0 and NetProtect^®^ evaluated by I-ACT and WHO cone and tunnel tests. In total, 432 nets (144/brand) were evaluated using I-ACT and cone test. Olyset^®^ nets (132/144) that did not meet the WHO cone test threshold criteria (≥ 80% mortality or ≥ 95% knockdown) were evaluated using tunnel tests with threshold criteria of ≥ 80% mortality or ≥ 90% feeding inhibition for WHO tunnel and I-ACT. Pass rate of nets tested by WHO combined standard WHO bioassays (cone/tunnel tests) was compared to pass in I-ACT only by net brand and time after distribution.

**Results:**

Overall, more nets passed WHO threshold criteria when tested with I-ACT than with standard WHO bioassays 92% vs 69%, (OR: 4.1, 95% CI 3.5–4.7, p < 0.0001). The proportion of Olyset^®^ nets that passed differed if WHO 2005 or WHO 2013 LN testing guidelines were followed: 77% vs 71%, respectively. Based on I-ACT results, PermaNet^®^ 2.0 and NetProtect^®^ demonstrated superior mortality and non-inferior feeding inhibition to Olyset^®^ over 3 years of field use in Tanzania.

**Conclusion:**

Ifakara Ambient Chamber Test may have use for durability studies and non-inferiority testing of new ITN products. It measures composite bioefficacy and physical integrity with both mortality and feeding inhibition endpoints, using fewer mosquitoes than standard WHO bioassays (cone and tunnel tests). The I-ACT is a high-throughput assay to evaluate ITN products that work through either contact toxicity or feeding inhibition. I-ACT allows mosquitoes to interact with a host sleeping underneath a net as encountered in the field, without risk to human participants.

**Electronic supplementary material:**

The online version of this article (10.1186/s12936-019-2741-y) contains supplementary material, which is available to authorized users.

## Background

National malaria control programmes (NMCPs) must ensure that all people living in malaria transmission areas are protected through the provision, nightly use and timely replacement of high quality long-lasting insecticidal nets (ITNs) and where appropriate, the additional application of indoor residual spraying (IRS) [1]. While it is assumed that all ITNs that have World Health Organization (WHO) prequalification listing last for 3 years, several ITN products are available that may vary in price as well as performance under local conditions [1–7]. Because ITNs are the primary means of malaria control, their durability, measured through physical integrity and bioefficacy against anopheline mosquitoes, needs to be accurately assessed in order to inform NMCPs of the most cost effective products and the correct interval for net replenishment campaigns [8].

Any ITN product is expected to retain its insecticidal activity (bioefficacy) for a minimum number of 20 standard washes or 3 years of use under field conditions as defined by the WHO [9]. However, the durability (years of functional life) of both existing and new net products under development is a crucial consideration. Despite mass distribution of ITNs, currently fewer than 50% of people living in malaria endemic areas are covered by one of the core malaria interventions: either ITNs or IRS [10]. Maximizing ITN access through the provision of the most long-lasting, and cost-effective products remains a critical concern, particularly as a number of countries have shown an increase in malaria in the past year (2016/2017) as investments in malaria control have plateaued [10].

For products within new intervention classes e.g. dual active ITNs, an Evidence Review Group (ERG) report to the WHO Malaria Policy Advisory Committee (MPAC) recommended specific guidance on the assessment of non-inferiority of products within a class [11]. A non-inferiority trial of an intervention aims to demonstrate that the test product is not worse than the comparator/reference by more than a pre-specified margin [12], known as the non-inferiority margin. For ITNs this margin relates to mortality or feeding inhibition. In recognition of the importance of ITN durability, the WHO recommended that once sufficient test and active comparator ITNs from large-scale field trials have been collected over 3 years of field use, a second set of two non-inferiority trials should be conducted to ensure that the test product continues to be non-inferior to the comparator/reference product for up to 3 years on both mosquito mortality and blood-feeding inhibition endpoints [13]. While this guidance recommended that non-inferiority trials should be conducted in experimental huts it was acknowledged that alternative methodology for non-inferiority testing including the ambient chamber test or the tunnel test should be explored.

The standard means of ITN bioefficacy evaluation is through cone bioassays, WHO tunnel tests and experimental hut evaluations [14]. The cone test is a contact assay where mosquitoes are held in proximity to the ITN and mosquito knockdown (KD60) and 24-h mortality are recorded after 60 min and 24 h, respectively. The tunnel test uses a live animal as a bait (rabbit or guinea pig), so mosquitoes are able to exercise host-seeking behaviour, and ITN efficacy is assessed by measuring mosquito mortality and blood feeding inhibition [15–17]. Experimental huts are small scale field (phase II) testing assays used to evaluate ITNs that meet laboratory (phase I) testing criteria [8, 18]. Huts are built in areas with high densities of target mosquito species and are designed to resemble small local housing but have features to retain mosquitoes that enter huts such as window traps and baffles [19]. Volunteers sleep underneath the ITNs and wild mosquitoes attempt to feed and interact with the ITNs in the same way as they would in local homes. Both mortality and feeding inhibition are key outcome parameters, which translate to personal and community protection from malaria [20].

However, all assays have some limitations, which need to be considered when assessing bioefficacy of ITNs. WHO cone tests may underestimate the induced mortality of irritant insecticides, as mosquitoes do not settle on treated nets [21]. Indeed, comparatively higher mortality is often measured in experimental hut studies of ITNs where mosquitoes make repeated contacts with treated nets as they try to feed on human volunteers sleeping under nets. In the WHO tunnel test, the live host used as bait is not the preferred host for the strongly anthropophilic Afro-tropical vector *Anopheles gambiae* sensu stricto (s.s.) [22] and may overestimate feeding inhibition. Alternatively, mosquitoes must be reared by feeding them on small mammals to select them for a preference to these non-preferred hosts, which is both expensive and of animal welfare concern. Experimental hut bioassays are the gold standard for ITN and IRS evaluation, but wild mosquito populations are often seasonal and have high temporal heterogeneity requiring substantial replication to ensure adequate power to detect true effect differences between products [23].

Therefore, presented here is the first evaluation of a new standardized semi-field assay: the Ifakara Ambient Chamber Test (I-ACT) assay. The assay was used to evaluate the bioefficacy of whole ITNs that were returned from the field in a longitudinal durability study. This study measured the bioefficacy of used (field-aged) ITNs using the I-ACT assay and standard WHO durability testing bioassays (cone and tunnel tests). The proportion of nets passing WHO criteria by standard methods and I-ACT was compared. The aim was to demonstrate the utility of this new assay for measuring bioefficacy of different ITN products and to explore its applicability for non-inferiority testing of new ITN products [11]. Further work comparing the I-ACT and experimental hut evaluations of ITNs will be reported separately.

## Methods

### Study design

Bioefficacy tests were conducted as part of a 3-year prospective project (the ABCDR-Attrition, Bioefficacy, Chemical residual, Damage and Resistance project) to assess of the useful life of three brands of ITNs in Tanzania [24]. The main design characteristics of each bioassay performed in this study are presented in Table 1. The study involved two experiments. In the first experiment, ITNs efficacy, measured by cone bioassay and I-ACT were compared. In the second experiment, ITNs bioefficacy measured by WHO tunnel test and the I-ACT was compared. The overall pass/fail rate for each net brand by year were examined following the criteria outlined in both 2005 and the 2013 World Health Organization guidelines for evaluation of long lasting nets [14, 25]. The pass rate of each product by standard WHO methods and I-ACT was compared.

**Table 1:** Design characteristics of the WHO cone test, WHO tunnel test and I-ACT

Particular	WHO cone test	WHO tunnel test	Ifakara Ambient Chamber test (I-ACT)
Diagram			
Endpoints measured	Knock down (KD 60), 24-h mortality	12-h mortality, 24-h mortality, feeding inhibition	12-h mortality, 24-h mortality, feeding inhibition
Infrastructure required	Temperature controlled room, boards, aspirators, cones, insect rearing facilities	Temperature controlled room, tunnel, aspirators, insect rearing facilities, animal rearing facilities	Ambient or temperature controlled chambers, temperature controlled holding room, aspirators, insect rearing facilities
Bait used	No	Rabbit, guinea pig	Human
Mosquitoes per net	80	100	30
Exposure time	3 min	12–15 h	12 h
Holding time	24 h	None	24 h
Time to conduct including preparation	25 h	16 h	26 h
Surface area exposed to mosquitoes	78 cm^2^	625 cm^2^	145,200 cm^2^
Useful for durability monitoring	Measures presence of insecticide	Measures mortality and feeding inhibition on a small section of net	Measures the functional efficacy of nets under user conditions
Useful for non-inferiority testing	Not suitable for some ITN products	Works for all ITNs	Works for all ITNs

### Mosquito rearing

Mosquitoes used during testing were laboratory-reared fully pyrethroid susceptible 3–8 days old female adult *An. gambiae* s.s. (Ifakara strain, Njage 1996) reared following standard methods [26].

### Mosquito nets

All mosquito nets used in this study came from a 3-years prospective longitudinal follow-up study between 2013 and 2016 (ABCDR Project) conducted in eight districts of Tanzania. Net samples were randomly selected using net codes from master list from the three surveys conducted between October and December 2014 (year 1), October–December 2015 (year 2) and October–December 2016 (year 3). The detailed description of the ABCDR Project has been reported previously [24]. Three net brands were used for this study: (1) Olyset^®^ net (permethrin incorporated into polyethylene fibres @ 1000 mg/m^2^), (2) PermaNet^®^ 2.0 net (deltamethrin coated on polyester fibres @ 55 mg/m^2^) and (3) Netprotect^®^ net (deltamethrin incorporated into polyethylene fibres @ 63 mg/m^2^). All nets were rectangular, white, double sized (190 cm length × 180 cm width × 150 cm height) and recommended by WHO [27]. All nets were used in the I-ACT as found i.e. with damage due to wear and tear. In the first experiment, to compare between cone test and I-ACT, a total of 432 nets (144 per net brand) were evaluated and results compared. In the second experiment to compare between tunnel test and I-ACT, nets those failed to meet cone test threshold criteria in the first experiment were assessed using WHO tunnel test (132 nets) and their results were compared with those from I-ACT.

### Ifakara Ambient Chamber Test (I-ACT)

**Fig. 1:**
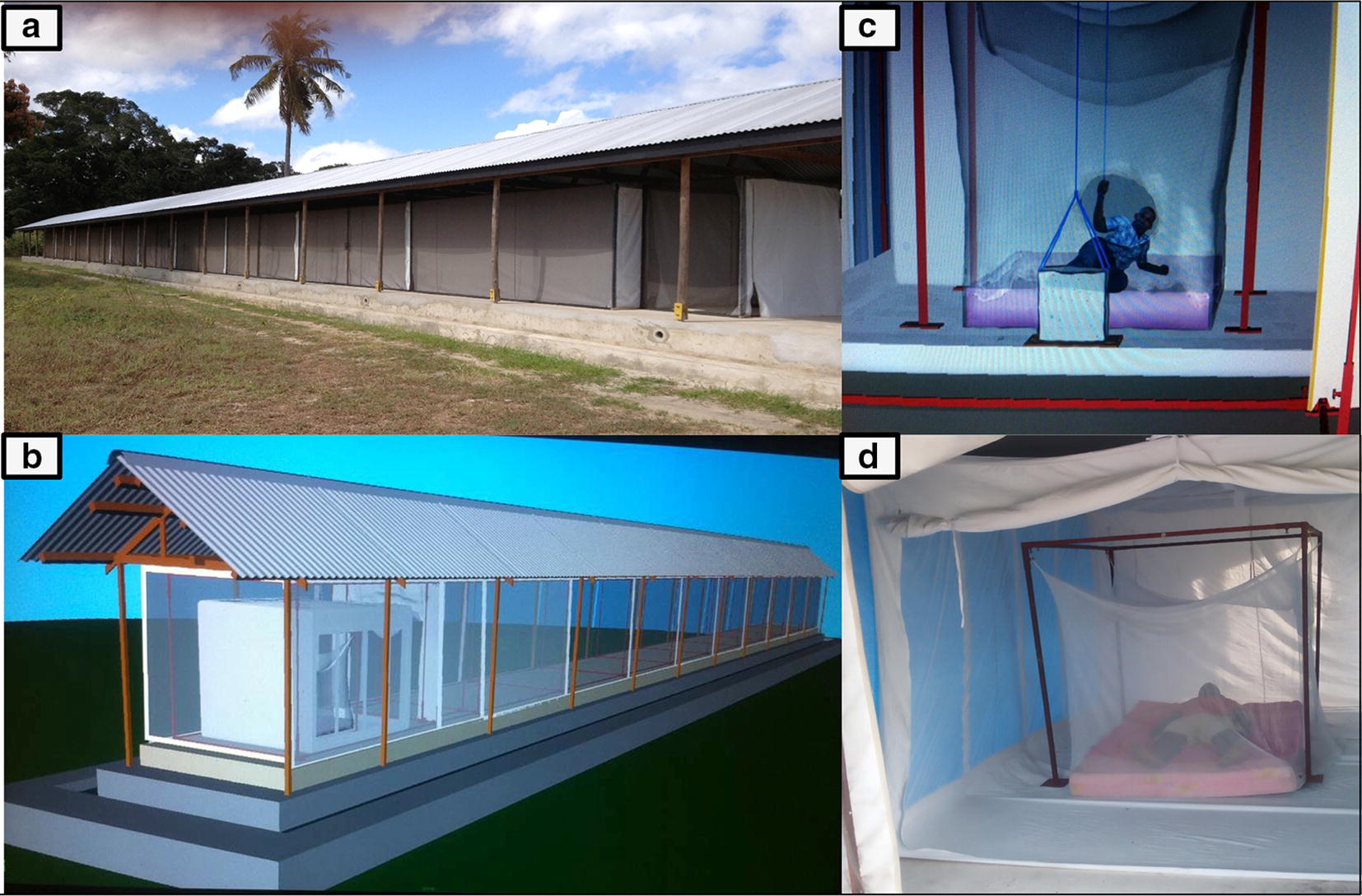
The I-ACT. Ifakara Tunnel situated at Bagamoyo branch of IHI (**a**). Net covered tunnel divided into 10 individual compartments each containing netted cage 2 × 2 × 5 m (**b**). The volunteer releases mosquitoes by opening the lid of the holding boxes while beneath the tested net (**c**). A human volunteer sleeps underneath the ITN (**d**)

This is a 50 m long, 3 m wide and 2.1 m high steel tube frame construction (Fig. 1a) covered by durable UV resistant polyurethane coated netting with an overlaid polyurethane sheet to minimize wind so that bioassays are conducted in still air (as would occur in a house). The structure is constructed upon a concrete base surrounded by a water channel to prevent entry by ants and spiders that eat mosquitoes during the conduct of experiments. The tunnel sits beneath a simple beamed wooden frame supporting a corrugated steel roof to allow work to continue in all weather conditions. The netted tunnel is divided into ten individual test chambers with interconnecting doors that are sealed by means of zips and Velcro to prevent mosquitoes moving from one test chamber to another. Each compartment contains a white netted chamber 5 m long, 2 m wide, and 2 m high that seals with a zip, in which the ITN is hung from a frame with a human volunteer sleeping underneath (Fig. 1b). At each end of the tunnel is an additional double door module to ensure no loss of laboratory-reared mosquitoes into the wild. Mosquitoes are released from the holding cages within each netted chamber by means of raising a netted cage from its removable wooden base. This is achieved by the technician in situ underneath his net pulling a nylon line attached to the mosquito release cage to elevate it (Fig. 1c). After the allotted experimental time period all mosquitoes within each of the compartments are recovered by mouth aspiration (for mosquitoes inside the net) and by a battery powered Prokopack aspirator (for mosquitoes outside the net but inside the compartment). This allows whole ITNs to be tested in a controlled ambient chamber test with a human host sleeping beneath (Fig. 1d) to measure the protective efficacy (both personal protection measured by feeding inhibition and community protection measured by mosquito mortality) under user conditions. The design of the chambers allows 100% recovery of released mosquitoes that improves precision of the data, and experiments can be conducted year-round.

Each of the ten testing chambers was randomly assigned one whole net (with wear and tear as found after use for 1, 2 or 3 years) from one of the three net brands using a random number generator. Two chambers were used each night as negative control with untreated SAFI Net (A to Z, Tanzania) holed with six holes 4 × 4 cm (Additional file 1) i.e. two holes on each large side and one hole on each small side (hole surface area of 96 cm^2^) according to WHO guidance [9]. One adult volunteer per chamber slept underneath the nets from 21:00 to 06:30 h and collected mosquitoes in the mornings. Each volunteer was fixed to the same chamber for the duration of the experiment. On each night of experiment, each volunteer hung the tested net on the bednet frame and tucked it underneath the mattress (between 28 and 35 cm of each net was tucked). At 21:00 h, each volunteer released 30 mosquitoes within the chamber but outside of the ITN by opening the mosquito release cage while remaining beneath their test net. The following morning, at 06:30 h, mosquitoes inside the net were collected first using a mouth aspirator and mosquitoes outside the net but within the chamber (floor and walls) were collected using a 6 V battery driven mechanical aspirator (Prokopack). A study supervisor checked the start and finish of the experiment and intermittently spot checked that the volunteers were in position overnight to ensure good conduct of the experiment. All collected mosquitoes were placed in paper cups and scored as dead-fed, alive-fed, dead-unfed, alive-unfed after which mosquitoes were held for 24 h in the laboratory with access to 10% sugar solution at 27 °C ± 2 and 80% ± 10 relative humidity and scored again. After every experimental night, all tested nets were taken out and chambers were aired and bed sheets were washed daily to prevent any carry-over insecticide residue. Each net sample was tested on two consecutive nights (fixed to a chamber and volunteer) to improve the precision of the estimation of performance of each net. Outcome measures were 24 h mortality and blood feeding inhibition. Nets that induced ≥ 90% blood-feeding inhibition and/or ≥ 80% mortality were regarded as meeting WHO efficacy criteria. Data were discarded and test repeated if control mortality exceeded 10% or control blood-feeding success was less than 50%. A Standard Operating Procedure for conducting Ifakara Ambient Chamber Test is provided as an Additional file 2.

### Cone tests

Cone tests were conducted following WHO guidelines [8] to determine the bioefficacy of insecticides on sampled netting pieces. For each of the sampled whole nets, after completion of the I-ACT, four 30 cm × 30 cm sub-samples were cut from positions 2, 3, 4 and 5 from each net sample (Additional file 3). Cone bioassays were held at a 60° vertical angle on the netting sub-samples [28]. *Anopheles gambiae* s.s., aged 3–5 days old, were exposed for 3 min after which they were held for 24 h with access to 10% sugar solution at 27 °C ± 2 and 80% ± 10 relative humidity. The numbers of mosquitoes knocked down after 60 min (KD60) and dead at 24 h post-exposure were recorded. A sub-sampled net that caused ≥ 95% KD60 and/or ≥ 80% 24 h mortality in the cone test was regarded as meeting WHO efficacy criteria. Tests, where control mortality at 24 h exceeded 10%, were discarded and repeated.

### WHO tunnel test

WHO tunnel tests were conducted following WHO guidelines [8] to assess the efficacy of netting sub-samples that failed to meet the WHO threshold criteria for cone test (95% KD60 and/or 80% 24 h mortality). The surface area of the sample netting accessible to mosquitoes was approximately 625 cm^2^ (25 × 25 cm) with nine holes cut in the net, each 1 cm in diameter: one at the centre of the square and the other eight holes were equidistant and located 5 cm from the border. The sampled net piece was inserted on a cardboard frame and positioned across the tunnel, one-third of the length of the tunnel. A total of 100 sugar-starved *An. gambiae* s.s. aged 5–8 days were released in the long section of the glass tunnel at 18:00 h. A rabbit was used as a bait and positioned on the other side of the net so that mosquitoes must pass through the holed net to access the bait and feed. The following morning, between 06:00 and 09:00 h, mosquitoes were removed (separately from each section of the tunnel) using a mouth aspirator, counted, scored as alive or dead, blood fed or unfed after which they were held for 24 h with access to 10% sugar solution at 27 °C ± 2 and 80% ± 10 relative humidity. The main outcome measures were 12 h mortality measured in the morning after the experiment and blood feeding inhibition [14]. The 24 h mortality was also recorded as a secondary outcome [25]. Nets that caused ≥ 90% blood-feeding inhibition and/or ≥ 80% mortality was regarded as meeting WHO efficacy criteria [25]. Tests were discarded if control mortality at 24 h exceeded 10% or control blood-feeding success was less than 50%.

### Data management and analysis

A sample size calculation for generalized linear mixed effects models (GLMMs) through simulation [12] in R statistical software 3.02 http://www.r-project.org [13] was performed for the semi-field experiments to detect a difference between the nets of 5% mortality (half the smallest anticipated effect size). Simulations were performed using an estimated mosquito mortality of 70% with the Olyset^®^ and 80% for the PermaNet^®^ 2.0 and NetProtect^®^. With 44 replicates tested on two occasions with an inter-observational variance of 0 for the chamber (controlled environment) and 0.1 for individual and 0.1 for the night of observation based on the variance of the random effects observed in a pilot study. Power was estimated at > 90% with a density of 30 mosquitoes per chamber per night using 1000 simulations.

Data were collected on standardized data collection forms and double entered into Microsoft Excel. Data were cleaned and analysed following a predefined analysis plan using STATA 14.1 (Stata Corp., College Station TX, USA) with significance level of ≤ 0.05 for rejecting the null hypothesis. Descriptive statistics were used to present the comparison of proportion of nets passing WHO threshold criteria as measured by each method. Generalized linear mixed models (GLMM) with a binomial error distribution and logit link function were used to analyse the main outcome measures from cone, tunnel and I-ACT (mortality and blood feeding) as well as the proportion of nets passing WHO criteria in order to detect differences between the two evaluation methods. Net brand, age of net and bioassay method were fitted as fixed effects while date was fitted as a random effect to account for repeated testing of individual nets. Several GLMMs were run for each comparison (with interactions) and the final model selected was that with the lowest Akaike’s Information Criterion (AIC). Residuals were plotted using histogram, qnorm plots and comparison with fitted values to ensure appropriateness of the model selection and testing if the residuals are normally distributed. Odds ratio (OR) and 95% confidence interval were calculated for the differences between methods in each comparison.

In addition, non-inferiority between net products (PermaNet^®^ 2.0, Netprotect^®^ with Olyset^®^ as reference/comparator) measured by I-ACT was analysed using a paired t-test with a 90% confidence interval of the observed effect difference in the mortality and bloodfeeding inhibition rates to measure non-inferiority at a margin of 10% and data were presented for comparison using a Forest Plot [29].

## Results

### Comparison between cone test and I-ACT

The data presented in Table 2 show that a smaller percentage of nets passed WHO threshold criteria using cone test 62% (268/432) than passed using by I-ACT 97% (417/432) irrespective of brand and net age (cone test criteria ≥ 80% 24-h mortality and/or ≥ 95% KD60; I-ACT criteria ≥ 80% 24-h mortality).

**Table 2:** Percentage and number of nets (by brand and age) meeting the standard WHO 2013 threshold criteria by I-ACT and cone tests

Age of net	Year 1		Year 2		Year 3		Overall	
	Cone test	I-ACT	Cone test	I-ACT	Cone test	I-ACT	Cone test	I-ACT
Olyset Net	4% (2/49)	100% (49/49)	8% (4/48)	96% (46/48)	13% (6/48)	86% (42/48)	8% (12/145)	94% (137/145)
PermaNet 2.0	98% (47/48)	100% (48/48)	92% (44/48)	98% (47/48)	73% (35/48)	96% (46/48)	88% (126/144)	98% (141/144)
Netprotect	100% (47/47)	100% (47/47)	100% (48/48)	100% (48/48)	73% (35/48)	92% (44/48)	91% (130/143)	97% (139/143)
Overall	67% (96/144)	100% (144/144)	67% (96/144)	98% (141/144)	53% (76/144)	92% (132/144)	62% (268/432)	97% (417/432)

WHO 2013 pass/fail criteria: cone test: ≥ 95% KD60 and/or ≥ 80% 24 h mortality and I-ACT: ≥ 80% 24 h mortality and/or ≥ 90% blood feeding inhibition

Using cone bioassays, 8% (12/145) of Olyset^®^ nets, 88% (126/144) of PermaNet^®^ 2.0 nets and 91% (130/143) of NetProtect^®^ nets passed the cone test. When tested by the I-ACT, 94% (137/145) Olyset^®^, 98% (141/144) PermaNet^®^ 2.0 and 97% (139/143) NetProtect^®^ passed (Table 2). Table 3 shows that, overall, I-ACT measured higher 24 h mosquito mortality than cone test regardless of net brand (OR: 7.9, 95% CI 7.4–8.4, p < 0.0001). Disaggregated by brand the same trend was evident and I-ACT measured higher mortality than cone test: Olyset^®^ nets (OR: 17.8, 95% CI 16.3–19.5%; p < 0.0001), PermaNet^®^ 2.0 (OR: 2.1, 95% CI 1.8–2.3%; p < 0.0001) and Netprotect^®^ (OR: 3.6, 95% CI 3.2–4.1, p < 0.0001).

**Table 3:** Measurements of percentage 24 h mortality compared between WHO cone test and I-ACT by net brand and age

	24 h geometric mean % mortality (95% confidence interval)				Odds ratio	95% confidence interval	p-value
	Year 1	Year 2	Year 3	Overall			
Olyset^®^							
Cone test	19.4 (17.9–20.9)	7.2 (6.2–8.2)	34.1 (32.1–36.2)	20.2 (19.2–21.2)	1		
I-ACT	87.2 (84.1–90.3)	68.9 (64.5–73.4)	69.8 (65.4–74.3)	75.1 (72.5–77.6)	17.8	16.3–19.5	< 0.0001
PermaNet^®^ 2.0							
Cone test	93.5 (92.4–94.7)	85.3 (83.3–87.2)	83.2 (81.4–85.1)	87.4 (86.4–88.3)	1		
I-ACT	98.4 (97.7–99.1)	91.5 (88.8–94.2)	87.8 (84.2–91.5)	92.5 (90.9–94.1)	2.1	1.8–2.3	< 0.0001
Netprotect^®^							
Cone test	93.1 (92.1–94.2)	81.1 (78.9–83.3)	82.2 (80.1–83.7)	85.4 (84.4–86.4)	1		
I-ACT	99.1 (98.6–99.6)	96 (94.4–97.6)	89.1 (85.9–92.1)	94.6 (93.4–95.9)	3.6	3.2–4.1	< 0.0001
Overall							
Cone test	68.2 (66.6–69.8)	58 (56.2–59.8)	66.5 (65.1–67.9)	64.2 (63.3–65.2)	1		
I-ACT	94.9 (93.7–96.2)	85.5 (83.2–87.7)	82.2 (79.8–84.5)	87.4 (86.2–88.6)	7.9	7.4–8.4	< 0.0001

#### Comparison between WHO tunnel test and I-ACT

A total of 164 nets (132 Olyset^®^, 19 PermaNet^®^ net and 13 Netprotect^®^) did not meet the WHO threshold criteria for cone test and therefore went for WHO tunnel tests. Only bio-efficacy results of Olyset^®^ sampled nets were used for comparison with I-ACT to ensure an adequately replicated and paired comparison, because the majority of PermaNet^®^ 2.0 and Netprotect^®^ sub-sampled nets passed the cone test.

The overall proportion of Olyset nets meeting WHO thresholds in tunnel test using methods outlined in WHO 2013 guidance (mortality recorded the morning after bioassay) and I-ACT is shown in Table 4. In addition, further analysis based on WHO 2005 guidance (mortality recorded after 24 h holding) was also performed and included in the results for comparison. Overall, results from Table 4 shows, regardless of the WHO ITN testing guideline used, more Olyset^®^ nets passed when measured in I-ACT than in tunnel test (using 24-h mortality OR: 5.7, 95% CI 2.5–, p < 0.0001).

**Table 4:** Overall percentage of sampled Olyset^®^ nets that met the standard WHO threshold criteria as measured by I-ACT and tunnel test following both WHOPES 2013 and 2005 guidelines

	No. of nets	% pass WHO 2013 criteria (n = number passed)	% pass WHO 2005 criteria (n = number passed)	Odds ratio	95% confidence interval	p-value
Year 1						
Tunnel test	47	60% (n = 29)	72% (n = 33)	1		
I-ACT	47	100% (n = 47)	100% (n = 47)	19.9	2.5–159.1	0.005
Year 2						
Tunnel test	44	75% (n = 33)	77% (n = 34)	1		
I-ACT	44	95% (n = 42)	95% (n = 41)	6.1	1.2–29.3	0.025
Year 3						
Tunnel test	41	66% (n = 27)	71% (n = 29)	1		
I-ACT	41	78% (n = 32)	76% (n = 31)	2.1	0.7–6.2	0.187
Overall						
Tunnel test	132	67% (n = 91)	72% (n = 96)	1		
I-ACT	132	92% (n = 121)	91% (n = 120)	5.7	2.5–12.9	< 0.0001

Odds ratios are calculated on 2005 criteria

WHOPES 2005 pass/fail criteria: tunnel test: feeding inhibition and/or ≥ 80% 24 h mortality

WHOPES 2013 pass/fail criteria: tunnel test: ≥ 90% feeding inhibition and/or ≥ 80% 12 h mortality

I-ACT: ≥ 80% 24 h mortality and/or ≥ 90% blood feeding inhibition

**Fig. 2:**
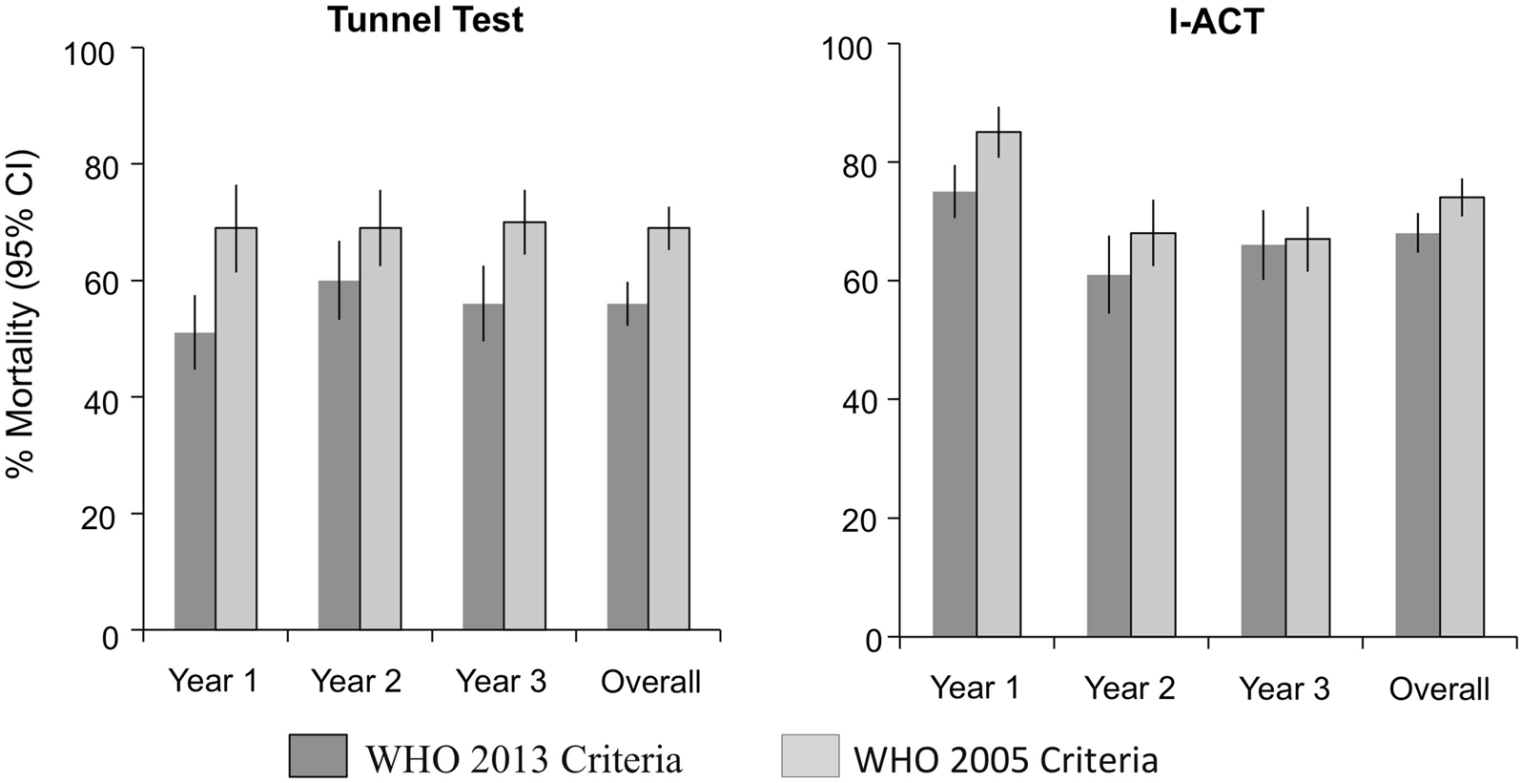
Mortality in susceptible *Anopheles gambiae* s.s. exposed to Olyset^®^ nets by year using the WHO tunnel bioassay (left panel) and the I-ACT (right panel) following WHOPES 2013 [59] and 2005 [25] guidelines for durability monitoring. Error bars indicate 95% confidence interval

**Fig. 3:**
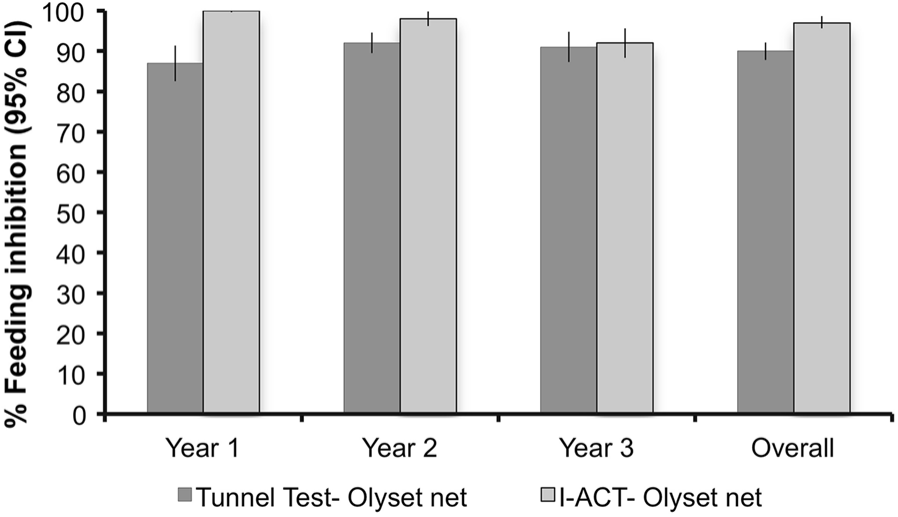
Mosquito blood feeding inhibition by year in susceptible *An. gambiae* s.s. exposed to Olyset^®^ nets using the WHO tunnel bioassay and I-ACT. Error bars indicate 95% confidence interval

Using either 12 h mortality or 24-h mortality, I-ACT recorded higher mortality than the tunnel test (Table 5). At 12 h, 64.1%, (95% CI 60.1–68.3%) vs 49.5% (95% CI 44.5–54.6%) (OR 1.7 (1.6–1.8), p < 0.0001) at 12 h and 71.2% (95% CI 67.7–74.9%) vs 64.4% (95% CI 59.8–69.4%) (OR 1.3 (1.2–1.4), p < 0.0001) at 24 h. For Olyset^®^ nets mortality was significantly higher measured after a 24-h holding period compared to the morning of collection in WHO tunnel test but not I-ACT (Fig. 2). Feeding inhibition (Fig. 3) of Olyset^®^ nets was also higher as measured by I-ACT (96.4%, 95% CI 94.7–98.1%) than tunnel test (88.9%, 95% CI 86.2–91.7%) (OR 3.6 (3.1–4.2), p < 0.0001). Similar trends were seen among the deltamethrin nets PermaNet^®^ 2.0 and NetProtect^®^ but data are not shown due to the imprecision of estimates from the low number of nets evaluated (19 and 13, respectively).

**Table 5:** Measurement of 12-h mortality, 24-h mortality and blood feeding inhibition compared between WHO Tunnel test and I-ACT for sampled Olyset^®^ nets through 3 years of field use

	Geometric mean (95% confidence interval)				Odds ratio	95% confidence interval	p-value
	Year 1	Year 2	Year 3	Overall			
Olyset							
% 12 h mortality							
Tunnel test	44.2 (36.5–53.5)	55.1 (47.8–63.5)	50.3 (42.2–60.1)	49.5 (44.9–54.6)	1		
I-ACT	73.2 (68.3–78.5)	56.1 (48.65–64.7)	63.4 (57.11–70.3)	64.1 (60.1–68.3)	1.7	1.6–1.8	< 0.0001
% 24 h mortality							
Tunnel test	61.4 (52.2–72.3)	64.7 (57.2–73.1)	67.7 (61.9–74.0)	64.4 (59.8–69.4)	1		
I-ACT	83.8 (78.9–89.1)	65.20 (59.56–71.4)	64.9 (59.15–71.2)	71.2 (67.7–74.9)	1.3	1.2–1.4	< 0.0001
% feeding inhibition							
Tunnel test	85.3 (80.0–90.8)	91.9 (89.2–94.7)	90 (84.8–95.6)	88.9 (86.2–91.7)	1		
I-ACT	99.6 (99.3–100)	98 (95.6–99.9)	91.3 (87.2–95.8)	96.4 (94.7–98.1)	3.6	3.1–4.2	< 0.0001

### Proportion of nets meeting the combined WHO methods

**Fig. 4:**
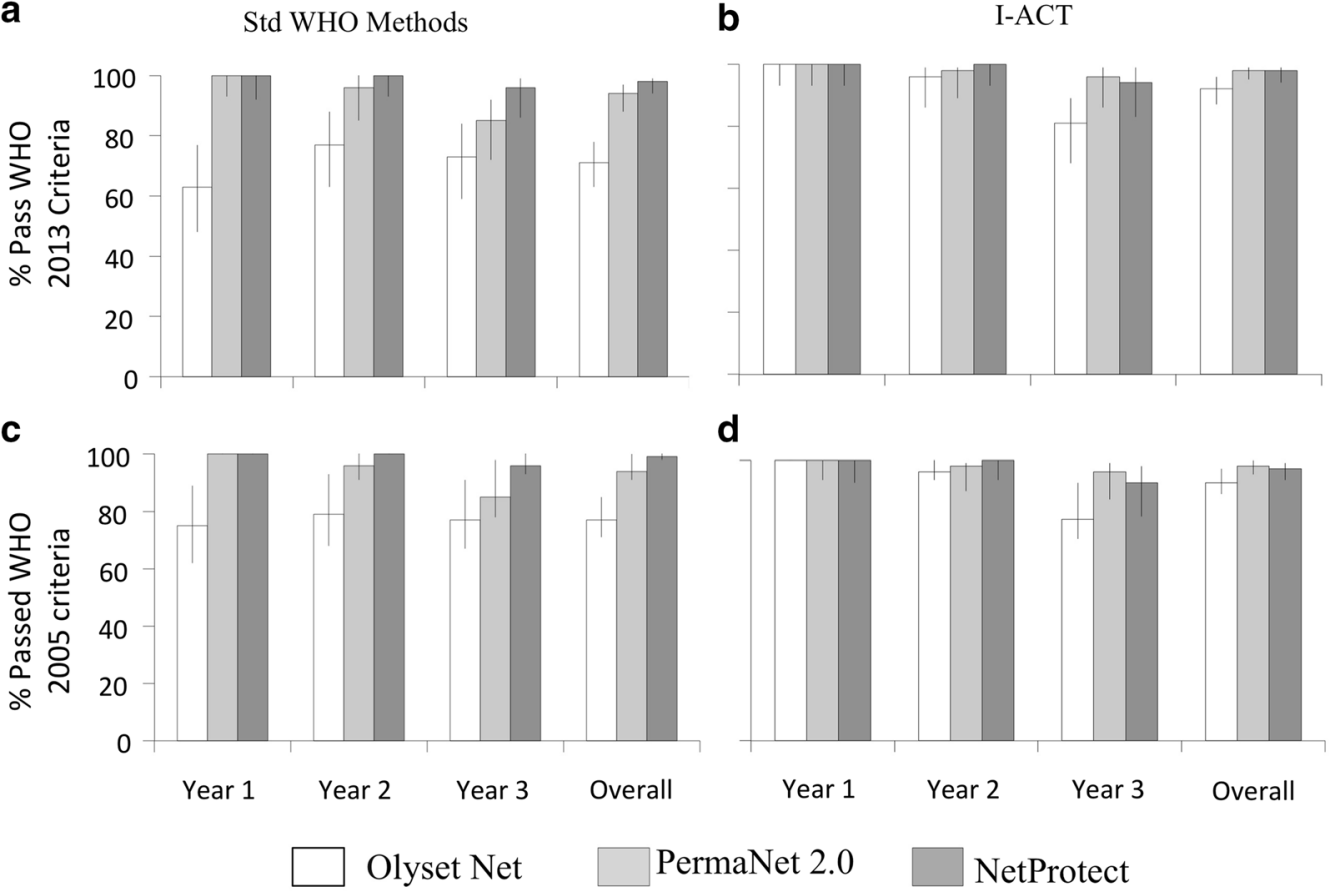
Percentage of ITNs by brand and age passing bioassay criteria following WHO 2013 and 2005 guidelines as measured by standard bioassays (**a**, **c**) vs I-ACT (**b**, **d**) against *An. gambiae* s.s. (Ifakara strain) fully susceptible to all classes of insecticides

Figure 4 shows that even after 3 years of field use PermaNet^®^ 2.0 and NetProtect^®^ killed a greater proportion of mosquitoes than Olyset^®^ resulting in a higher pass rate by WHO (combined cone/tunnel) methods, while there was less contract in the performance of the three products overall when tested using I-ACT. The data (Table 6) show that overall more nets passed WHO threshold criteria using I-ACT than using standard WHO (combined cone/tunnel) methods irrespective of brand and age (OR: 3.5, 95% CI 1.9–6.5, p < 0.0001). The proportion of nets passing using combined WHO methods agreed with I-ACT for NetProtect^®^ and PermaNet^®^ 2.0 but differed different for Olyset^®^ with 94% passing in I-ACT vs 77% by standard bioassays (OR 5.2, 95% CI 2.3–11.8, p < 0.0001). A second notable difference was that holding time was important in determining the pass rate of Olyset^®^ net with a significant increase in the proportion of Olyset^®^ that passed when 24 h mortality vs immediate mortality scoring was used: 77% (95% CI 69–83%) vs 71% (95% CI 62–78%) pass. A small and not significant difference was observed in the proportion of PermaNet^®^ 2.0 (94% vs 98%) and NetProtect^®^ (98% vs 97%) passing by either WHO methods or I-ACT methods, respectively.

**Table 6:** Difference in the proportion of nets passing WHO 2005 threshold criteria by combined WHO cone and Tunnel test methods compared to I-ACT for sampled Olyset^®^, PermaNet^®^ 2.0 and NetProtect^®^ nets

	No. of nets tested	% pass WHO 2013 criteria (n)	% pass WHO 2005 criteria (n)	Odds ratio	95% confidence interval	p-value
Olyset^®^						
WHO methods	145	71% (n = 103)	77% (n = 111)	1		
I-ACT	145	94% (n = 134)	94% (n = 137)	5.2	2.3–11.8	< 0.0001
PermaNet^®^ 2.0						
WHO methods	144	94% (n = 135)	94% (n = 135)	1		
I-ACT	144	98% (n = 141)	98% (n = 141)	3.1	0.8–11.8	0.092
NetProtect^®^						
WHO methods	143	99% (n = 141)	99% (n = 141)	1		
I-ACT	143	98% (n = 140)	97% (n = 139)	0.49	0.1–2.7	0.418
Overall						
WHO methods	432	69% (n = 379)	90% (n = 387)	1		
I-ACT	432	92% (n = 415)	97% (n = 417)	3.5	1.9–6.5	< 0.0001

Odds ratios are calculated using pass/fail with 24 h holding times for all tests

WHOPES 2005 pass/fail criteria: tunnel test: feeding inhibition and/or ≥ 80% 24 h mortality

I-ACT: ≥ 80% 24 h mortality and/or ≥ 90% blood feeding inhibition

### Non-inferiority of sampled net product

**Fig. 5:**
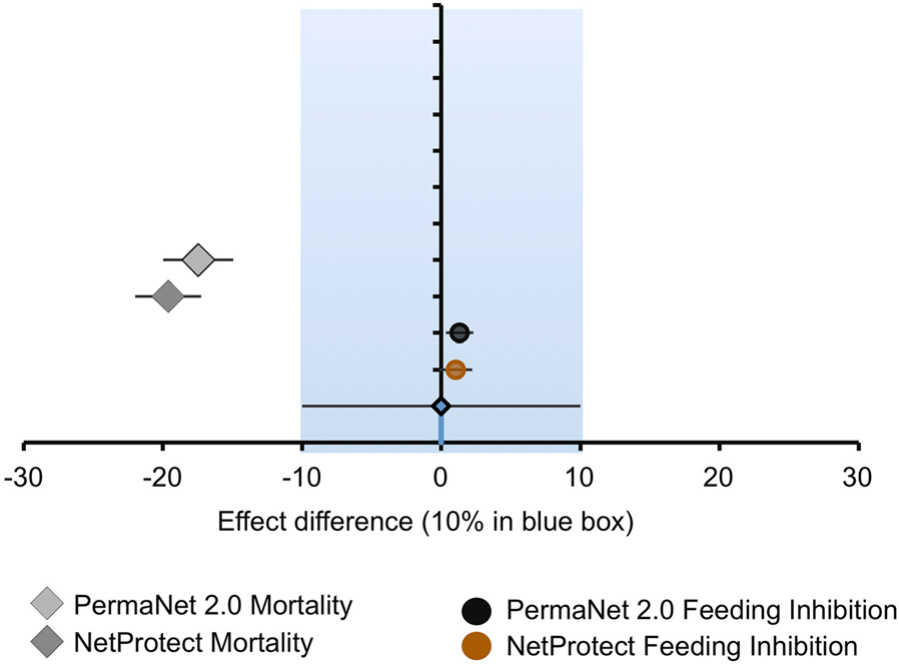
Non-inferiority of PermaNet 2.0 net and NetProtect combined 24 h mortality and feeding inhibition for 3 years of data with Olyset^®^ as the reference performed in the I-ACT using a 10% margin of non-inferiority

In order to measure non-inferiority of field aged nets Olyset^®^ was used as the reference net (active comparator for non-inferiority testing) and the other two nets were compared to it. Using I-ACT data it can be seen that overall, PermaNet^®^ 2.0 and Netprotect^®^ killed greater proportion of mosquitoes than Olyset^®^. Using a t-test of the effect difference between the products using the 24 h mortality endpoint with a margin of 10% of non-inferiority it can be seen that both PermaNet^®^ 2.0 and NetProtect^®^ were superior to Olyset^®^ (Fig. 5). It should be noted that the figure shows a negative value for superior products because the effect difference is calculated by subtracting the induced mortality of the test net from the reference net. However, using the feeding inhibition endpoint, both PermaNet^®^ 2.0 and NetProtect^®^ were non-inferior to Olyset using a 10% margin of non-inferiority. It can, therefore, be concluded that the three products are equivalent based on a combined mortality and feeding inhibition endpoints, as is current WHO practice.

## Discussion

This is the first ITN durability study to compare bioefficacy of ITNs using standard WHO bioassays (cone and tunnel tests) with data collected from whole nets tested using the I-ACT. A large numbers of three brands of nets returned from the field were evaluated in I-ACT to measure their protection to users sleeping underneath them in the presence of natural wear and tear. The I-ACT allowed high throughput (48 nets per brand per year) to give precise estimates of overall product efficacy. Data from the I-ACT for the deltamethrin nets PermaNet^®^ 2.0 and Netprotect^®^ that function through rapid knock down and mortality largely agrees with the standard WHO methods (cone/tunnel tests). However, for Olyset^®^ that functions through the prevention of feeding a greater proportion of nets passed using I-ACT than standard WHO methods (cone/tunnel tests).

It was observed that each of the three net brands showed lower efficacy measured by standard WHO bioassays compared to efficacy measured by I-ACT. This could be due to: (1) duration of exposure (3 min vs 12 h), (2) surface area of treated fabric presented to the mosquitoes (both standard WHO methods use 20 cm^2^ samples versus a whole net in the I-ACT) and (3) number of tarsal contacts with the ITN resulting in exposure to different dose of insecticide due to the presence of a human host under the net for the I-ACT. In cone test experiments, mosquitoes are exposed to tested ITN for only 3 min which may not allow the tested mosquitoes to exercise natural host-seeking behaviour with multiple contacts over the net surface resulting in a higher cumulative dose of insecticide. This has also been measured by other authors in studies to understand behavioural and physiological changes in mosquitoes in relation to responses to insecticides. A series of studies by Angarita-Jaimes and colleagues using a novel video-tracking system to quantify the behaviour of nocturnal mosquitoes attacking human hosts in the laboratory and in field observed that, both *An. gambiae* s.s. and *Culex quinquefasciatus* showed multiple contacts with bed nets when a human host was present [30], and this host seeking activity is lower for treated nets than in untreated nets [31, 32]. However, the I-ACT study demonstrated that these contacts were sufficient to kill or inhibit feeding among the majority of pyrethroid susceptible mosquitoes used in this study.

In addition, the findings add to existing data that shows that the cone test underestimates the bioefficacy of Olyset^®^ that contains Permethrin, a contact irritant pyrethroid [15, 17, 33, 34]. During cone tests, permethrin causes mosquitoes to minimize contact with the netting fibres and they may sometimes rest on the sides of the cone or cotton plug on the cone and avoid the insecticide and demonstrate frequent take offs from the net [28].

The tunnel test was developed as a consequence of the need to measure feeding inhibition of permethrin-treated nets [35] and has also shown some use in evaluating products that fail cone tests including chlorfenapyr products as it allows mosquitoes to exhibit flight and host seeking feeding behaviour in a natural simulated condition [36]. However, as with cone test, the tunnel test has some limitations. The overall pass rate (using 12 or 24-h mortality and blood feeding inhibition), as measured following both WHO 2005 and 2013 criteria, was lower compared to that measured by I-ACT. A possible explanation for this observation is that, the baits used in tunnel tests are rabbits that are not the preferred bait for *An. gambiae* s.s. that feeds almost exclusively on humans [22, 37]. Therefore, during standard tunnel test experiments, mosquitoes may be less responsive to non-preferred bait and remain in the releasing chamber throughout the exposure time without interacting with the ITN sample resulting in a lower cumulative exposure to insecticide. Additionally, using a whole net in the I-ACT killed more mosquitoes possibly due to the large surface area of insecticide available for mosquitoes to interact with. It is known from repellent testing that use of a non-preferred bait will overestimate repellent efficacy [38]. However, a similar number of PermaNet^®^ 2.0 and NetProtect^®^ passed the combined WHO tests and the I-ACT whereas fewer Olyset^®^ passed combined WHO tests indicating that the WHO tests are conservative and therefore unlikely to pass a product that is of low efficacy. As the I-ACT is a less conservative test it may have use for early screening of new insecticide treated nets including those with irritant insecticides or those that function through a mode of action other than rapid knockdown before more costly experimental hut tests.

The overall percentage of Olyset^®^ nets that passed tunnel test following WHO 2013 guidelines was marginally lower than when following WHO 2005 guidelines [25]. This suggests that reinstating the WHO 2005 pass/fail efficacy criteria may be justified to avoid missing products that are efficacious during early testing. This will also align tunnel test holding times with those of cone bioassays and experimental huts (24 h). It may also be justifiable when testing some products to hold mosquitoes for even longer than 24 h as some authors have done to measure the effects of slow acting insecticides [39, 40]. This simple pairwise test between the two guidelines demonstrated the usefulness of exploring the impact of holding time on the outcome of product tests.

The mode of action of insecticides used on ITNs is an important consideration when selecting bioassays. New products with modes of action different from pyrethroids (which are fast acting and neurotoxic) are coming to market and there is a need for a suitable means to bioassay them. An example is chlorfenapyr, which acts by disrupting metabolic respiratory pathways (oxidative phosphorylation) in the cells of mitochondria and that require the conversion of the active compound through metabolism [41]. The conversion is optimal at night and is maximized when mosquitoes are metabolically active i.e. during the active part of their circadian rhythm and flying during host seeking [42]. Cone tests are usually conducted during the day and take 3 min exposure time with no bait involved. Findings from two well conducted studies by Oxborough et al. and Ngufor et al. observed extremely low levels of mortality caused by chlorfenapyr compared to pyrethroids when assessed by cone test, but excellent effect against resistant mosquitoes when tested in experimental huts [39, 43]. These data again suggest that cone test may be best suited for fast acting non-irritant insecticides [35] and there is a need to be open to exploring new bioassays for new mode-of-action products. The higher pass rate of I-ACT compared to standard WHO tests may be useful when conducting “quick and dirty” tests for new products to avoid early “kill” of promising products because they are failing to pass bioefficacy criteria in phase I laboratory tests when they may prove highly efficacious in gold standard experimental hut tests (Phase II).

Ifakara Ambient Chamber Test may be useful in evaluating new products that function through either mortality or feeding inhibition. Tests are conducted at times when mosquitoes are metabolically active, and using the preferred host of Afro-tropical malaria vectors. The advantage of using the I-ACT is that nets are evaluated using mortality and feeding inhibition using just one test rather than having to perform the cone (for mortality) followed by the tunnel test bioassays (for feeding inhibition or mortality at night). Regarding the issue of precision in outcome measure estimates, the durability study performed here in the I-ACT used 30 mosquitoes per chamber per night of experiment and allowed large numbers of nets to be evaluated without exhausting the insectary which is always a concern when product testing. It is important to assess a large number of nets in durability studies to allow a sufficient sample of nets to be returned from the field to capture the large heterogeneity in product performance i.e. fabric integrity and insecticidal content, and using a random sampling framework that is large enough to avoid sampling bias such as the Hawthorne effect [44].

When the efficacy of ITNs was compared using standard WHO assays and I-ACT, it was seen that most of the tested nets were extremely effective against mosquitoes and passed WHO criteria of feeding inhibition and/or mortality using the pyrethroid susceptible *An. gambiae* s.s. (Ifakara) strain even after 3 years of use with natural damage and insecticide depletion from the field. This has also been shown by other research in Tanzania [34, 45, 46]. Many of the tested nets were damaged. The median hole surface area was 459 cm^2^ in Olyset, 295 cm^2^ in Permanet and 152 cm^2^ in NetProtect in year 3, which means that most surviving nets were in the “damaged” category, but remained highly protective.

In addition, a simple non-inferiority test was conducted using WHO criteria to evaluate the effect of difference between products for mortality and feeding inhibition. Olyset^®^ was used as the reference product (first in class or active comparator) against which the two other brands (second in class, test product or innovator product) were compared since it is the standard of care in Tanzania. PermaNet^®^ 2.0 and Netprotect^®^ were non-inferior compared to Olyset^®^ on the feeding inhibition endpoint and superior to Olyset^®^ on the 24-h mortality endpoint when measured in the I-ACT. The WHO passes a product based on a combination of mortality and feeding inhibition, and based on these criteria, PermaNet^®^ 2.0 and Netprotect^®^ were non-inferior to Olyset^®^ based on data for three-year durability. This was also seen with WHO bioassays: Olyset^®^ demonstrated lower mortality and similar feeding inhibition to PermaNet^®^ 2.0 and Netprotect^®^ when tested using cone tests and tunnel tests. Estimates of efficacy from the sample of 144 nets per brand were very precise and a 10% effect difference in mortality could be observed. However, it is unlikely that 144 nets per brand could be cost effectively evaluated in experimental huts. A comparison study between Ifakara experimental huts and the I-ACT using 24-h mortality and feeding inhibition outcome measures is in progress (Moore et al., pers. commun.) and will show how I-ACT and gold standard experimental huts compare for non-inferiority evaluation of ITNs. This is important since experimental huts are used to measure entomological correlates of the epidemiological effectiveness i.e. the public health benefit of interventions [47].

Therefore, the I-ACT could prove useful for testing insecticidal materials that can provide a high throughput option for evaluating *functional bioefficacy* of ITNs i.e. the true protection as a function of damage and bioavailability of insecticide in durability studies. Functional bioefficacy i.e. incorporating insecticidal effectiveness has also been suggested by WHO’s Malaria Policy Advisory committee to be included for net durability assessment [48]. While the methods presented here may not be useful for operational durability monitoring they may be useful for consideration in WHO “Phase 3” community field assessments of ITNs.

In this new assay, recapture of released mosquitoes is 99% so 30 mosquitoes were consistently “captured” every night I every chamber which is unlikely to be the case in standard experimental hut studies [45, 49–57]. Experimental hut studies rely on wild mosquitoes entering the hut, and the nightly number of mosquitoes captured is highly variable and consequently substantial replication is required to obtain adequate precision to estimate true effect differences between products [23]. As mosquito densities fluctuate due to seasonality in rainfall it is useful to have a whole net assay that is not dependent on field populations of mosquitoes that may limit the windows of opportunity to conduct tests with adequate mosquito densities to achieve power. Whole net bioassays where the interaction between insecticide and fabric integrity is measured are important for selecting between products or ranking their durability [48]. Bioassays that assess only the insecticidal bioefficacy of a net sample may favour poor quality nets that tear easily reducing user protection and consequently user acceptance, which will eventually lead to the user discarding the net [58].

The experimental hut bioassay that simulates domestic conditions and allows nets to be tested against wild mosquitoes is the definitive test of ITN efficacy [43]. This study had several limitations. Firstly, I-ACT uses laboratory-reared mosquitoes, which means it relies on laboratory strains that may have different resistance mechanisms to those locally or limited genetic diversity. Secondly, the I-ACT test is a more expensive infrastructure to establish compared to small WHO cones and WHO tunnel glass chambers, requires space and it is immovable. The assay must be conducted in climate-controlled chambers or in areas with suitable ambient conditions to conduct the tests. In contrast standard WHO cones and tunnel chambers which can be taken anywhere and tests conducted provided the environment is set to standard conditions for conducting tests. The I-ACT needs to be compared to experimental hut tests, but it did agree well with findings of standard WHO methods using pyrethroid susceptible mosquitoes. Evaluations of ITNs with pyrethroid resistant strains as well as using dual active ITNs will be reported in subsequent publications.

Based on the data here presented, the overnight I-ACT may be a bridge between the lab and the field. Data agreement with standard WHO testing methods was excellent, with high sensitivity and specificity. It allows mosquitoes to host seek during the active phase of the circadian rhythm, and have multiple contacts with treated netting in a more realistic way. It uses the preferred human host but allows laboratory-reared mosquitoes to be used. This improves safety for human volunteers because laboratory-reared mosquitoes are disease free and allows sufficient numbers of mosquitoes to be released to reach the power needed to conduct precise comparisons of product performance.

## Conclusion

Findings from this study showed that, I-ACT can be used for high throughput evaluation of whole nets from ITN durability studies. The new assay may provide useful additional information and could act as a link between lab tests and field experiments measuring composite bioefficacy and net physical integrity with both mortality and feeding inhibition endpoints. For the three products evaluated in this study the bioassay agreed with standard WHO tests for deltamethrin products and measured higher pass for the permethrin-treated nets than standard WHO tests. I-ACT allows mosquitoes to interact with a preferred host sleeping under a net as it would be encountered in the field using a standard number of mosquitoes released to improve the precision of efficacy estimates and safety of human participants.

## Additional files

**Additional file 1.** Location of holes on the deliberately holed SAFI net.

**Additional file 2.** A standardized operating procedure for conducting experiments to measure feeding inhibition and mortality of different net products using the I-ACT.

**Additional file 3.** Sampling patterns of the net for cone bioassay and tunnel test.
