# Supplementary material for: Comparing the new Ifakara Ambient Chamber Test with WHO cone and tunnel tests for bioefficacy and non-inferiority testing of insecticide-treated nets
Source: Malar J. 2019 Apr 30;18:153. doi: 10.1186/s12936-019-2741-y (PMC6492396; doi:10.1186/s12936-019-2741-y)
Supplement: Supplementary file 1 — Additional file 1. Location of holes on the deliberately holed SAFI net. [file 12936_2019_2741_MOESM1_ESM.pdf]

Title	Ifakara ambient chamber LN test

1.0 Objective

The purpose of this document is to provide step-by-step instructions for conducting experiments to measure feeding inhibition and mortality of nets for the IT using the Ifakara ambient chamber LN test (Figure 4).

2.0 Definitions

2.1 Abbreviations

SOP: Standard operating procedure
IN: Insectary
LN: Long lasting insecticide treated nets
IT: Insecticide testing group

2.2 Glossary:

For the purposes of this SOP we classify dead mosquitoes as

– Mosquitoes that show no movement or,
– Mosquitoes that cannot stand due to the loss of > 3 limbs or either wing.

For the purposes of this SOP we classify alive mosquitoes as

- Mosquitoes that can stand and fly in a coordinated manner.

2.4 Outcome measures

- Blood feeding inhibition
- Mortality after exposure

3.0 Equipment

- 1 x Broom
- 1 x Spider brush
- 1 x Mouth aspirator
- 10 x prokopacks
- 20 x prokopack cups, correctly labeled (2 per chamber, labeled inside or outside)
- 10 x prokopack "extension poles"
- 10 x Netted "release boxes"

4.0 Methods

4.1 Recruitment of volunteers will be performed and informed consent taken.

4.2 Weekly screening of volunteers will be performed and recorded.

4.3 Volunteers will report to work at 18.30 and one volunteer will be allocated to each compartment by the study supervisor.

4.4 Each volunteer will clean their compartment using a spider broom to remove predators such as ants and spiders and prepare their sleeping space by placing a clean sheet on the mattress.

4.5 Supervisors will hang a test or control LN in each compartment over the frame as per LN test net rotation sheet. They will place a fully charged prokopack within each chamber.

4.6 At 20.30 volunteers will enter their sleeping space beneath the LN.

4.7 A supervisor will place a netted "release box" of 30 nulliparous 3-8 old uninfected, actively host seeking mosquitoes into each compartment with its removable wooden base and place adjacent to the foot side of the bed (outside the bed but inside the tunnel chamber).

4.8 A supervisor will then check that each of the two collection cups for the prokopack are correctly labelled with date, chamber number, number of the LN, and collection location: INSIDE, OUTSIDE.

4.9 The supervisor will attach the mosquito cage to the nylon rope suspended over a lever such that when volunteers pull the rope from inside the sleeping space, mosquitoes will be released freely **(Figure 1*).***

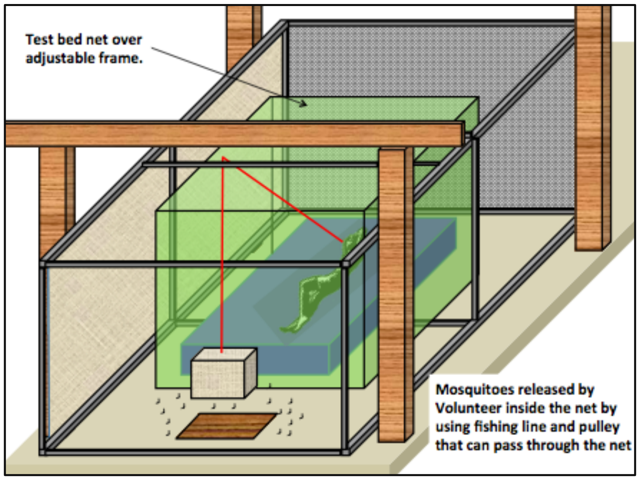

Figure 1: Mosquitoes released by Volunteer inside the net (© Courtesy of Jason Moore, IHI)

4.10 Supervisors must check that all the preparations are done correctly in each tunnel chamber and check that all interconnecting doors are correctly sealed by means of zips and Velcro to prevent mosquitoes moving from one test chamber to another ready to start the experiment.

4.11 At 20:50 the study supervisor performs the final check that all the volunteers are in place, that volunteers are comfortable and that there is no light in any tunnel chamber.

4.12 Supervisors must make sure that all the outside doors are closed to ensure no loss of mosquitoes into the wild.

4.13 At 21:00 hours, supervisor shows a sign to inform all the volunteers to release the mosquitoes by gently pulling down the lever simultaneously and mosquitoes will be released by means of raising a netted cage from its removable wooden cage.

4.14 Volunteers must remain in their sleeping space under their LN until 06:30 hours.

4.15 At 06:30 hours, the volunteers will collect mosquitoes from within their LN using the prokopack with the collection cup labelled INSIDE working from the bottom right corner of the net in a clockwise direction, working from the bottom up and then down (Figure 2*).* This must be completed in 3 minutes.

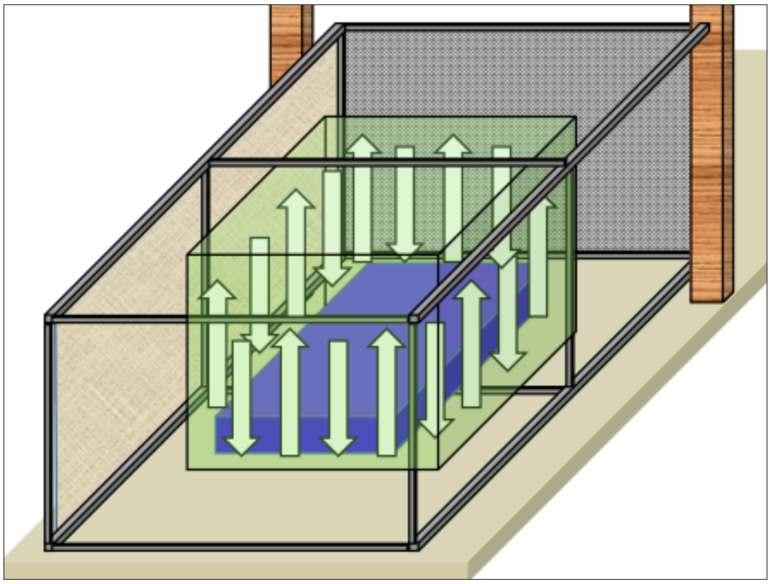

Figure 2: Direction for collection of Mosquitoes inside the bednet. (© Courtesy of Jason Moore, IHI)

4.16 When the inside of the LN has been cleared of mosquitoes, the collection cup must be closed using its lid.

4.17 The prokopack may then be switched off once the collection cup is sealed with its lid.

4.18 Each volunteer will then attach the collection cup labelled OUTSIDE and switch on the prokopack.

4.19 Each volunteer will collect knocked down and dead mosquitoes from the floor starting at the right middle side of the sleeping space and working from right to left clockwise until they have checked the entire floor, taking care not to stand on any dead mosquitoes.

4.20 When the floor has been cleared and the volunteer has reached the right middle of the sleeping space after checking the floor, the collection cup must be sealed with its lid.

4.21 The prokopack may then be switched off once the collection cup is sealed. Each volunteer will then attach the prokopack extension to assist in reaching the top of the chamber, then switch on the prokopack and unseal the collection cup to continue collecting.

4.22 Each volunteer will proceed to collect mosquitoes resting on the walls of the tunnel working from the bottom right corner of the chamber in a clockwise direction, working from the bottom up then down (Figure 3).

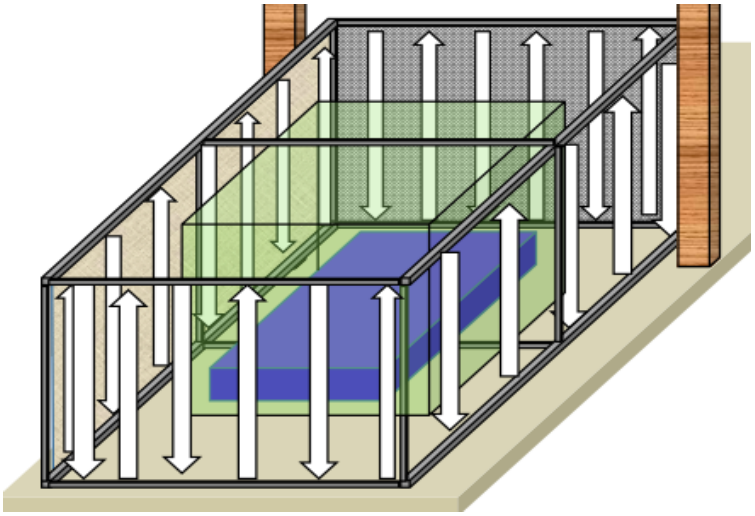

Figure 3. Direction for collection of Mosquitoes inside tunnel chamber (© Courtesy of Jason Moore, IHI)

4.23 When the inside of the chamber has been cleared of mosquitoes, the collection cup must be closed with its lid.

4.24 The prokopack may then be switched off once the collection cup is sealed.

4.25 Once all volunteers have completed this procedure they are free to leave using the double door exit point, ensuring that only one door is open at a time to minimize the chance of any mosquito escape.

4.26 Study technicians will then collect the prokopack cups from each chamber, checking for any remaining mosquitoes that the volunteers may have missed.

4.27 All collection cups are taken to the test centre holding room.

4.28 Mosquitoes are scored as fed alive, unfed alive, fed dead, unfed dead, and remain in their catch paper cups.

4.29 A piece of glucose soaked cotton wool is placed on the netting of the cup and the holding room maintained at 27±2°C and 80±10% humidity for 24 hours.

4.30 Technicians return to the tunnel and put on disposable gloves.

4.31 Technicians return each net to its clear plastic bag for storage and return them to storage.

4.32 After 24 hours mosquitoes are scored as fed alive, unfed alive, fed dead, unfed dead on the data sheet.

4.33 All cups containing mosquitoes are placed into the -4℃ freezer for a minimum of 10 minutes to kill the mosquitoes and they are disposed of.

4.34 After each test all sheets are washed and new cups used.

4.35 At the end of each week the study supervisor must take the data sheets to the study director for verification.

5.0 Ifakara ambient chamber test (I-ACT) structural design

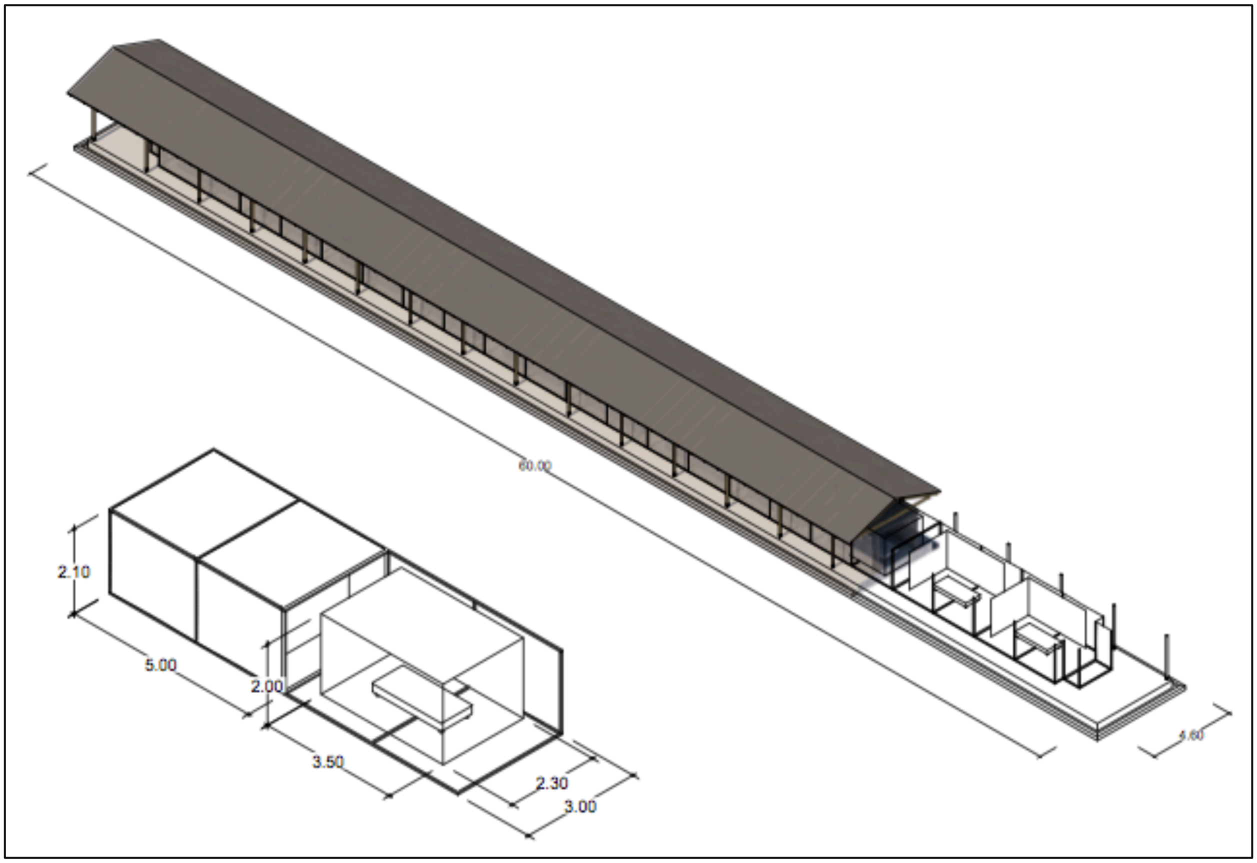

Figure 4. Ifakara Ambient Chamber Test design (© Courtesy of Samuel Ackerman , IHI)

____________________________ END of SOP____________________________
