# Supplementary figures and images for: Comparing the new Ifakara Ambient Chamber Test with WHO cone and tunnel tests for bioefficacy and non-inferiority testing of insecticide-treated nets
Source: Malar J. 2019 Apr 30;18:153. doi: 10.1186/s12936-019-2741-y (PMC6492396; doi:10.1186/s12936-019-2741-y)

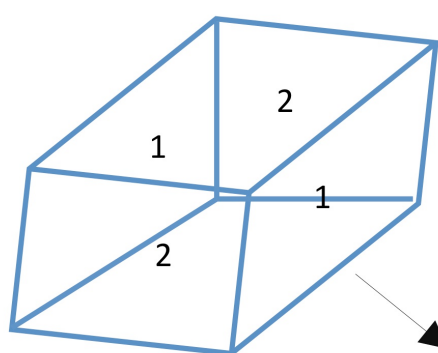

Rectangular net

Side panels of a net

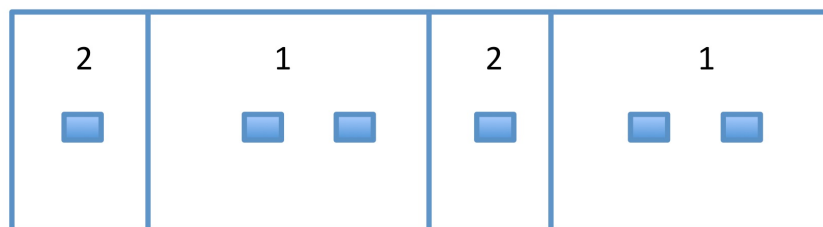

Figure 2

Supplement: Supplementary file 2 — Additional file 2. A standardized operating procedure for conducting experiments to measure feeding inhibition and mortality of different net products using the I-ACT. [file 12936_2019_2741_MOESM2_ESM.pdf]

Rectangular net

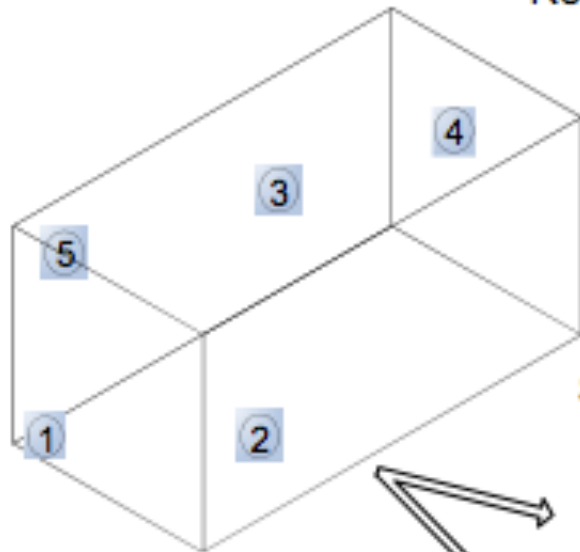

Side panels

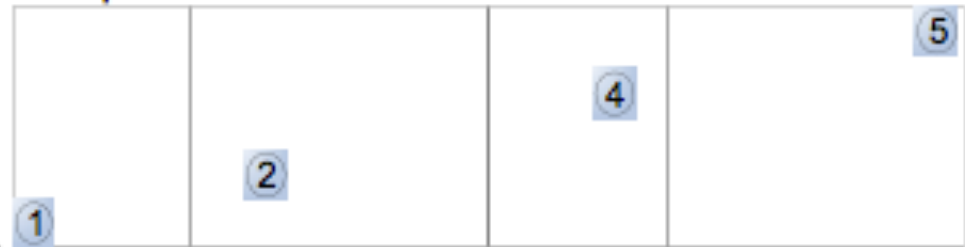

Roof panel

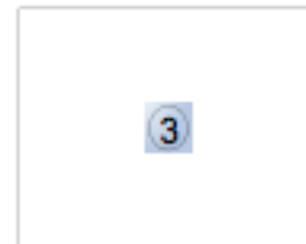

SOM 2(b)

Supplement: Supplementary file 3 — Additional file 3. Sampling patterns of the net for cone bioassay and tunnel test. [file 12936_2019_2741_MOESM3_ESM.pdf]
